# Crystal Growth and Design of Disk/Filament ZnO-Decorated 1D TiO_2_ Composite Ceramics for Photoexcited Device Applications

**DOI:** 10.3390/nano11030667

**Published:** 2021-03-08

**Authors:** Yuan-Chang Liang, Wei-Cheng Zhao

**Affiliations:** Department of Optoelectronics and Materials Technology, National Taiwan Ocean University, Keelung 20224, Taiwan; qaz5263153@gmail.com

**Keywords:** synthesis, crystal feature, composite ceramics, photoresponse

## Abstract

Disk- and filament-like ZnO crystals were decorated on one-dimensional TiO_2_ nanostructures (TiO_2_–ZnO) through various integrated physical and chemical synthesis methods. The morphology of the ZnO crystals on TiO_2_ varied with the chemical synthesis method used. ZnO nanodisks decorated with TiO_2_ nanorods (TiO_2_–ZnO–C) were synthesized using the chemical bath deposition method, and ZnO filament-like crystals decorated with TiO_2_ nanorods (TiO_2_–ZnO–H) were synthesized through the hydrothermal method. Compared with the pristine TiO_2_ nanorods, the as-synthesized TiO_2_–ZnO composites exhibited enhanced photophysiochemical performance. Furthermore, because of their fast electron transportation and abundant surface active sites, the ZnO nanodisks in the TiO_2_–ZnO–C composite exhibited a higher photoactivity than those in the TiO_2_–ZnO–H composite. The morphology and crystal quality of the ZnO decoration layer were manipulated using different synthesis methods to realize disk- or filament-like ZnO-decorated TiO_2_ composites with various photoactive performance levels.

## 1. Introduction

TiO_2_ nanorods, which are one-dimensional (1D) oxides, have wide applicability in various photophysiochemical devices [1,2,3,4]. The crystalline quality, composition, and morphology of TiO_2_ nanorods influence their efficiencies [5,6]. TiO_2_ nanorods can be synthesized through various physical and chemical methods. Hydrothermal crystal growth is preferred for synthesizing free-standing TiO_2_ nanorod arrays because this method enables large-area crystal growth, facile process parameter control, and the use of flexible substrate materials [7,8,9]. ZnO crystals, such as nanosheets or nanoplates, are promising components for photoexcited nanodevices [10,11,12]. Numerous synthesis methods, including chemical vapor deposition [13], evaporation [14], chemical bath deposition [15], and hydrothermal methods [10], have been proposed for fabricating sheet-, plate-, or disk-like ZnO crystal composites. In contrast to other methods, hydrothermal and chemical bath deposition methods can be used for fabricating homogeneous ZnO crystals with a large area distribution over substrates because these methods are simple, have low cost, require a low growth temperature, and provide a high yield.

The nanosheet-decorated 1D nanorod hierarchical structure is used in the fabrication of various photosensitive nanodevices because this composite structure exhibits high light utilization efficiency and a large effective surface area and can facilitate rapid charge transfer and the efficient collection of photogenerated carriers. For example, 1D TiO_2_-nanosheet Bi_2_S_3_ composites exhibit excellent photoelectrochemical performance; this is attributable to the efficient charge transfer ability in the system [16]. Moreover, the WO_3_–ZnO composite exhibits superior photocatalytic performance, which is associated with efficient charge separation in the system [17]. Furthermore, the photoexcited properties of ZnO nanorod arrays have been substantially improved through the decoration of tin sulfide nanosheets [18]. On the basis of the aforementioned attributes, 2D oxide semiconductors cross-linked on 1D oxide nanorods form a heterogeneous hierarchical structure, which is a promising nanoplatform for improving photoactive performance through efficient charge separation and transfer in the composite system. Notably, band alignment has revealed the presence of a type-II heterojunction between TiO_2_ and ZnO for various morphologies, such as TiO_2_ nanorods–ZnO nanorods and TiO_2_ nanowire–ZnO nanoparticles, which exhibit superior photoactive performance to their constituents [19,20]. However, limited studies have been conducted on the synthesis of TiO_2_–ZnO branched hierarchical composites, such as 1D TiO_2_–ZnO sheets (or disks). Furthermore, the related ZnO morphology and crystal-quality-influenced photoactive performance of TiO_2_–ZnO composites have not been systematically investigated. In this study, TiO_2_–ZnO composites comprising 1D TiO_2_ and disk- or filament-like ZnO nanostructures were fabricated by integrating the method assisted by a sputtering-grown ZnO seed layer and various chemical ZnO synthesis methods. The crystal characterization of the ZnO nanostructures on TiO_2_ nanorods was controlled by varying parameters of chemical bath deposition and hydrothermal crystal growth. The as-synthesized TiO_2_–ZnO composites exhibited superior photophysiochemical performance to pristine TiO_2_ nanorods. This result was associated with band matching between ZnO and TiO_2_ together with the rationally designed dimensionality-dependent heterojunctions for promoting photoactivity. Tuning the morphology and crystal quality of as-synthesized TiO_2_–ZnO composites systems through suitable chemical synthesis methods with controllable sputtering ZnO seed layer quality is a promising approach for fabricating 1D TiO_2_–ZnO disk (or filament) composites. Such composites are used for fabricating desirable photoactive devices.

## 2. Experiments

**Figure 1:**
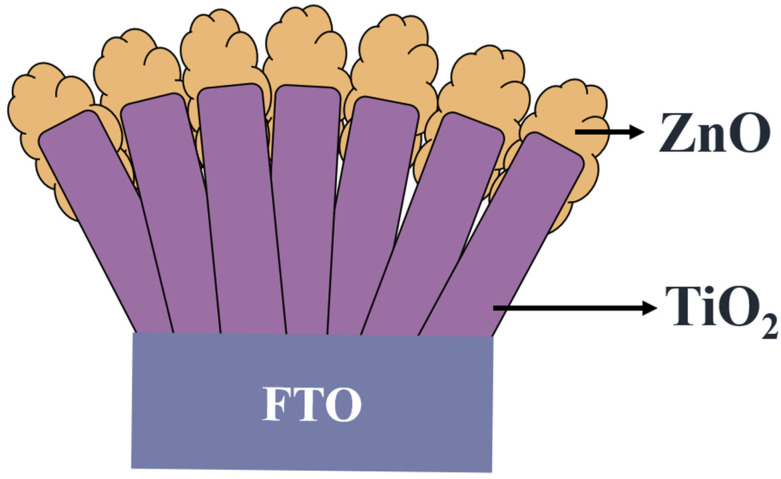
Schematic diagram of the TiO_2_–ZnO composite sample.

TiO_2_ nanorod arrays were grown on clean fluorine-doped tin oxide (FTO) substrates via a hydrothermal growth method. The detailed hydrothermal growth parameters of the TiO_2_ nanorod arrays have been described elsewhere [4]. For growing chemical-route-derived ZnO crystallites on the TiO_2_ nanorod template to form TiO_2_–ZnO composite structures, the ZnO seed layer was deposited onto the TiO_2_ nanorod template by radio-frequency sputtering (named TiO_2_–ZnO–S). A ZnO ceramic disc (99.999% Pure, 3.0 inch diameter) was used as the target for sputtering the ZnO seed layer. Herein, the argon and oxygen gas flow rates were set at 20 and 5 sccm, respectively. The ZnO seed layer deposition was performed at 90 W for 1 h at room temperature. In this study, two sets of TiO_2_–ZnO composite structures were prepared. The solution of 0.3 g of Zn(NO_3_)_2_ 6H_2_O, 0.14 g of hexamethylenetetramine (HMT, C_6_H_12_N_4_), and 0.028 g of sodium citrate (Na_3_C_6_H_5_O_7_) was mixed with 100 mL of DI water and stirred for 10 min. For chemical bath deposition (CBD), the prepared solution was further transferred to CBD apparatus and then kept in a water bath at a temperature of 90 °C for 4 h. The as-synthesized TiO_2_–ZnO composite structure via this approach was named TiO_2_–ZnO–C. On the other hand, the same reactant solution was transferred into 20 mL capacity Teflon enclosed with a stainless steel bottle for hydrothermal crystal growth. The reaction was conducted at 90 °C for 4 h. The as-synthesized product herein was named TiO_2_–ZnO–H. Figure 1 shows the schematic configuration of the as-synthesized composites in this study.

The surface and microstructures of the samples were investigated by scanning electron microscopy (SEM, Hitachi S-4800, Tokyo, Japan) and transmission electron microscopy (HRTEM; JEOL JEM-2100F, Tokyo, Japan). The crystalline phases of the samples were characterized by X-ray diffraction (XRD; D2 PHASER, Karlsruhe, Germany) using Cu Kα radiation. The absorption spectra of the samples were obtained using a diffuse-reflectance mode via an ultraviolet–visible spectrophotometer (UV–vis; Jasco V750, Tokyo, Japan). The photoelectrochemical (PEC) properties of the samples were obtained by an AutoLab electrochemical workstation (BioLogic SP150, Seyssinet-Pariset, France). Electrochemical impedance spectra (EIS) were measured on the same workstation with a saturated Ag/AgCl electrode. The sample was used as the working electrode. The counter electrode is Pt wire. The electrolyte contains 0.5 M Na_2_SO_4_. The photocatalytic activity of the samples was evaluated by the degradation of 10 mL of aqueous solution of methyl orange (MO; 5 × 10^−5^ M) dye with a 100 W Xe lamp as the light source. The photocatalyst sample size is 10 mm × 10 mm.

## 3. Results and Discussion

**Figure 2:**
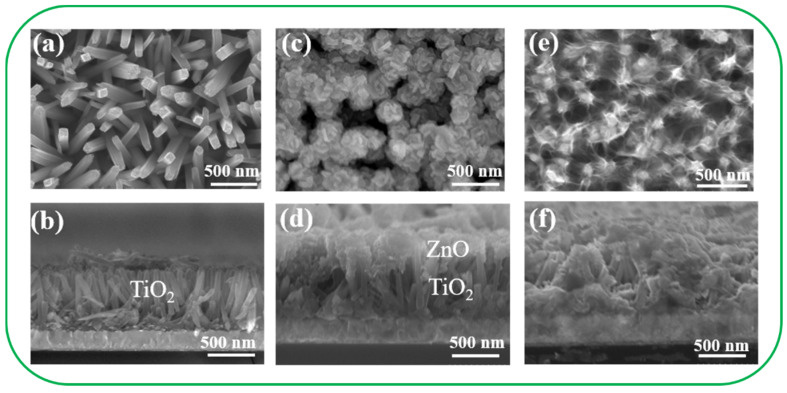
SEM images of various samples: (**a**,**b**) TiO_2_; (**c**,**d**) TiO_2_–ZnO–C; (**e**,**f**) TiO_2_–ZnO–H.

Figure 2a,b illustrates SEM micrographs of hydrothermally derived TiO_2_ nanorod templates. The TiO_2_ nanorods exhibited a square facet morphology, which is the expected growth habit for the tetragonal crystal structure. The TiO_2_ nanorod template exhibited smooth side facets. Furthermore, the cross-sectional view of the TiO_2_ nanorod template demonstrates a well-aligned feature on the fluorine-doped tin oxide substrate. The free-standing TiO_2_ nanorods had lengths and diameters of approximately 0.8–1.2 μm and 50–90 nm, respectively. Figure 2c,d illustrates the morphology of the TiO_2_ nanorod template decorated with CBD-derived ZnO nanodisks (TiO_2_–ZnO–C). The ZnO nanodisks were grown in a staggered manner on the TiO_2_ nanorods, which resulted in a branched morphology. The diameter of the ZnO nanodisks varied from 100 to 250 nm, with the thickness ranging from 30 to 50 nm. Notably, the ZnO nanodisks had a hexagonal shape corresponding to the wurtzite ZnO hexagonal crystallographic structure. The formation of hexagonal ZnO nanodisks was attributed to growth suppression on the (002) facet, which leads to the formation of six-fold symmetric hexagonal nanoplates [21]. The aggregation of numerous interlocked thin disk structures provides abundant spaces between primary ZnO disks filled with TiO_2_ nanorods, which may facilitate sufficient surface area exposure in the reaction environment. Figure 2e,f displays SEM micrographs of the TiO_2_ nanorod template decorated with hydrothermally derived ZnO filaments (TiO_2_–ZnO–H). Most ZnO aggregates were formed on the top regions of the TiO_2_ nanorods, and ZnO filaments extended outward to connect with each other. The thin ZnO filaments covered the TiO_2_ nanorod template like a net. SEM revealed that the ZnO crystals obtained through the two-step hydrothermal process and the CBD-derived ZnO crystals were distributed in the TiO_2_ top region and interweaved with neighboring segments. The results revealed that the ZnO nanostructures synthesized through various chemical routes considerably affected the morphologies of the TiO_2_–ZnO heterogeneous composites.

**Figure 3:**
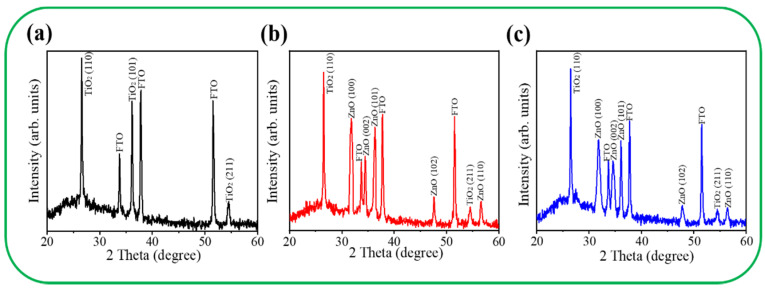
XRD patterns of various samples: (**a**) TiO_2_; (**b**) TiO_2_–ZnO–C; (**c**) TiO_2_–ZnO–H.

The reference XRD pattern of the pristine TiO_2_ nanorod template is shown in Figure 3a. In addition to FTO Bragg reflections, three clear diffraction peaks located at approximately 27.45°, 36.08°, and 54.32° correspond to the TiO_2_ crystallographic planes of (110), (101), and (211) (JCPDS No. 00-021-1276), respectively. Figure 3b,c presents the XRD patterns of the TiO_2_–ZnO–C and TiO_2_–ZnO–H composites, respectively. From the XRD patterns, several ZnO diffraction peaks were distinguished according to JCPDS No. 00-036-1451. Notably, the XRD results reveal (100) and (101) nonpolar planes dominated the crystallographic feature of the ZnO crystals decorated on the TiO_2_ nanorod templates. A similar nonpolar crystallographic-plane-dominated ZnO crystal feature has been reported in chemical-route-derived 2D ZnO sheets [10]. It should be mentioned that the positively Zn^2+^-terminated (002) facets and negatively O^2−^-terminated {002} polar surfaces of wurtzite ZnO are more reactive [15], and the fastest growth rate along the *c*-axis due to the higher surface energy of {002} planes is frequently observed in chemical-route-derived ZnO crystals [22]. In this study, the sodium citrate was introduced into the chemical solution process to serve as a structure-directing agent, and complexation between Zn^2+^ ions and citrate can ligand to suppress the ZnO crystal growth along the [001] direction [23]. The disclosed crystallographic feature of the chemical-route-derived ZnO crystals is consistent with the morphology observation from the SEM images. Comparatively, the ZnO Bragg reflections from the TiO_2_–ZnO–C are higher in intensity and narrower in peak width than those of the TiO_2_–ZnO–H, demonstrating a higher crystalline quality of the 2D ZnO crystals in the TiO_2_–ZnO–C.

**Figure 4:**
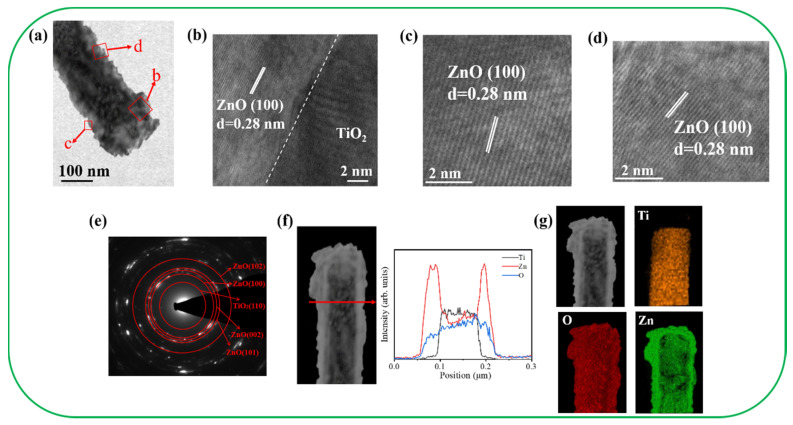
TEM analyses of TiO_2_–ZnO–C. (**a**) Low-magnification TEM image. (**b**–**d**) HRTEM images taken from various regions of the sample. (**e**) Selected area electron diffraction (SAED) pattern taken from several composite nanostructures. (**f**) EDS line-scanning profiles across the sample. (**g**) Element mapping images of the sample.

Figure 4a illustrates the morphology of the TiO_2_–ZnO–C composite rod. The diameter of the rod was approximately 120 nm. The ZnO crystals displayed in Figure 4a were grown on a TiO_2_ surface in a staggered manner. The decoration of the ZnO crystals on TiO_2_ resulted in the generation of irregular surface edges on the composite. The high-resolution (HR) TEM images (Figure 4b–d) of the local regions of the composite rod display the interface of the TiO_2_/ZnO and ZnO regions. A sharp interface was observed between TiO_2_ and ZnO. The remarkable lattice fringes with ordered arrangements and an interplanar spacing of 0.28 nm corresponded to the hexagonal ZnO(100) plane. The HRTEM results indicated that the TiO_2_–ZnO–C composite nanostructure exhibited a highly crystalline structure. Figure 4e illustrates the selected area electron diffraction (SAED) pattern of several TiO_2_–ZnO–C composite rods. The distinct spots arranged in concentric rings could be attributed to the hexagonal ZnO (100), (002), (101), and (102) planes and the rutile TiO_2_ (110) plane. The SAED results agreed with the X-ray diffraction results, which indicated that the crystalline TiO_2_–ZnO–C composite rods were formed through the proposed combined hydrothermal–chemical bath methodology. Figure 4f illustrates the spatial distribution of the Ti, Zn, and O elements across the TiO_2_–ZnO–C composite rod. This distribution was obtained using the TEM–energy-dispersive spectroscopy (EDS) line-scan profiling method. The variation of elemental intensity profiles indicated that Ti was mainly confined within the inner area of the composite rods and that Zn was distributed around the TiO_2_ nanorod template. Figure 4g illustrates the high-angle dark-field TEM image and the corresponding EDS elemental mapping images. The results revealed that Ti was perfectly filled in the inner area of the composite structure. Furthermore, the outer region of the composite structure clearly revealed the Zn signal. O was homogeneously distributed over the composite structure. The compositional analysis results revealed rational Zn, Ti, and O distribution in the TiO_2_–ZnO–C composite structure.

**Figure 5:**
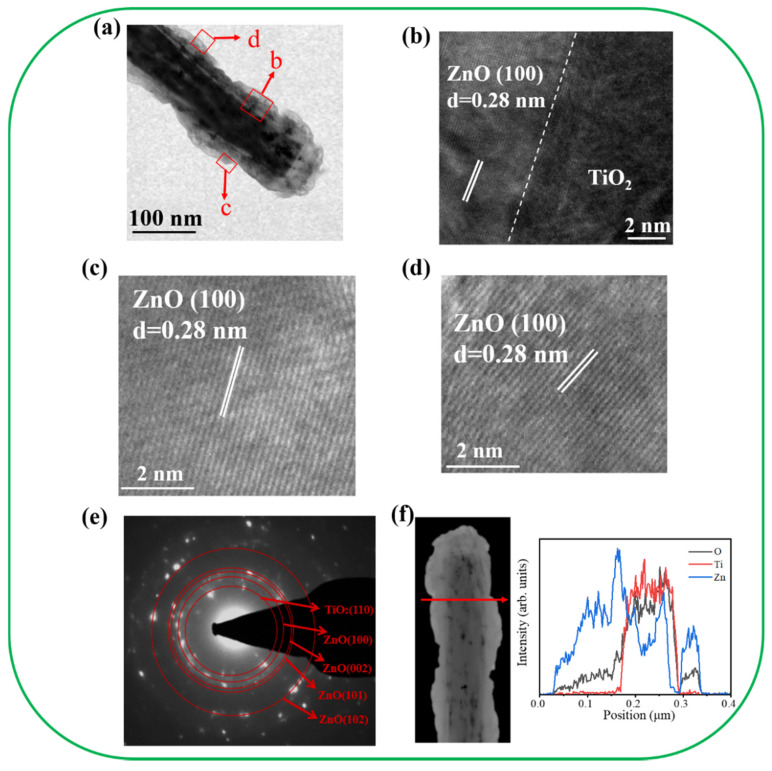
TEM analyses of TiO_2_–ZnO–H (**a**) Low-magnification TEM image. (**b**–**d**) HRTEM images taken from various regions of the sample. (**e**) SAED pattern of several composite nanostructures. (**f**) EDS line-scanning profiles across the sample.

Figure 5a depicts the morphology of the TiO_2_–ZnO–H composite rod. Feather-like ZnO crystals were visibly grown on the TiO_2_ nanorod template, indicating the formation of a composite structure. Figure 5b–d presents the HRTEM images of the local regions of TiO_2_/ZnO and ZnO of the TiO_2_–ZnO–H. The ordered fringe spacing of 0.28 nm matches well with the interplanar spacing of the ZnO (100) plane, revealing the crystalline feature of the decorated ZnO. Figure 5e displays the SAED pattern of several TiO_2_–ZnO–H composite rods. Numerous visible spots arranged in centric patterns were observed. The concentric rings were ascribed to diffractions from the rutile TiO_2_ (110) plane and the hexagonal ZnO (100), (002), (101), and (102) planes. The SAED analysis reveals the crystalline nature of the TiO_2_–ZnO–H. Figure 5f exhibits the EDS line-scan profiling spatial distribution of the Ti, Zn, and O across the TiO_2_–ZnO–H composite rod. The results herein indicate the two steps hydrothermal growth process is feasible for preparing a heterogeneous structure of TiO_2_/ZnO.

**Figure 6:**
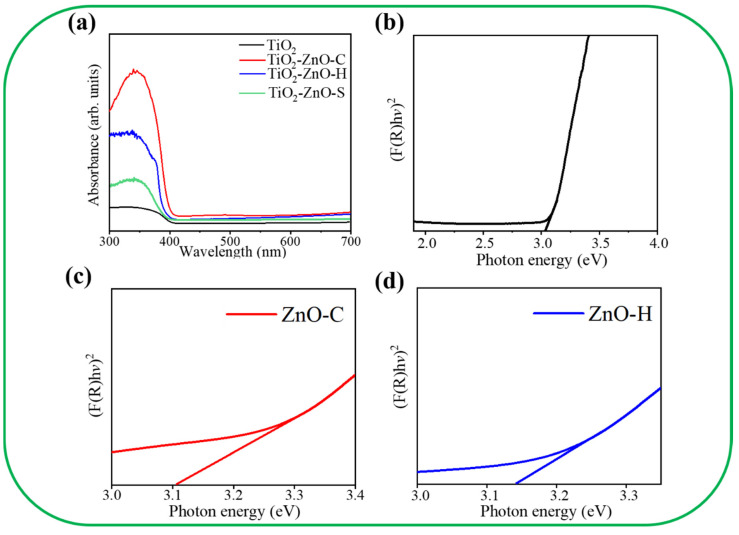
(**a**) Optical absorbance spectra of various samples. (**b**) Band gap of the TiO_2_. (**c**) Band gap of the ZnO nanodisks. (**d**) Band gap of the ZnO nanosheets.

Figure 6a shows the absorption spectra of various samples. A strong absorption edge at approximately 410 nm appears in the absorption spectrum of the TiO_2_ nanorod template. In Figure 6b, the bandgap energy of the TiO_2_ template is evaluated to be approximately 3.03 eV. After the ZnO nanostructures were grown on the surfaces of the TiO_2_ nanorod template via CBD or hydrothermal growth, the absorption edge of the TiO_2_–ZnO composites shows a slightly red shift in comparison with that of the pristine TiO_2_ nanorod template. In contrast, the pristine TiO_2_ nanorod template demonstrated a lower light-harvesting efficiency in comparison with the TiO_2_–ZnO composite structures from the absorption analysis. This is in agreement with recent work on TiO_2_ nanorod arrays/ZnO nanosheets heterostructured photoanodes, which also demonstrates that the TiO_2_–ZnO composite shows excellent light absorption ability than the pristine TiO_2_ nanorod [24]. Furthermore, TiO_2_–ZnO–C displays the highest light-harvesting ability among various samples. Figure 6c,d presents the bandgap energy evaluation of pristine ZnO crystals synthesized via CBD and hydrothermal growth, respectively. The detailed band gap energy evaluation process has been described elsewhere [25]. Notably, the ZnO crystals synthesized via the CBD route demonstrate a lower bandgap energy of 3.11 eV than that of the hydrothermally derived ZnO crystals. The different crystal qualities and features of the ZnO crystals synthesized via various chemical routes might result in the observed bandgap energy difference herein. The observations herein are supported by the work on the ZnO nanosheets/nanodisks synthesized by using ethyl cellulose and cetrimonium bromide as the capping and structure-directing agents. In that work, the as-synthesized ZnO nanodisks have a smaller bandgap energy in contrast to ZnO nanosheets, and this is attributed to the crystalline quality difference between the ZnO nanodisk and nanosheet [26].

**Figure 7:**
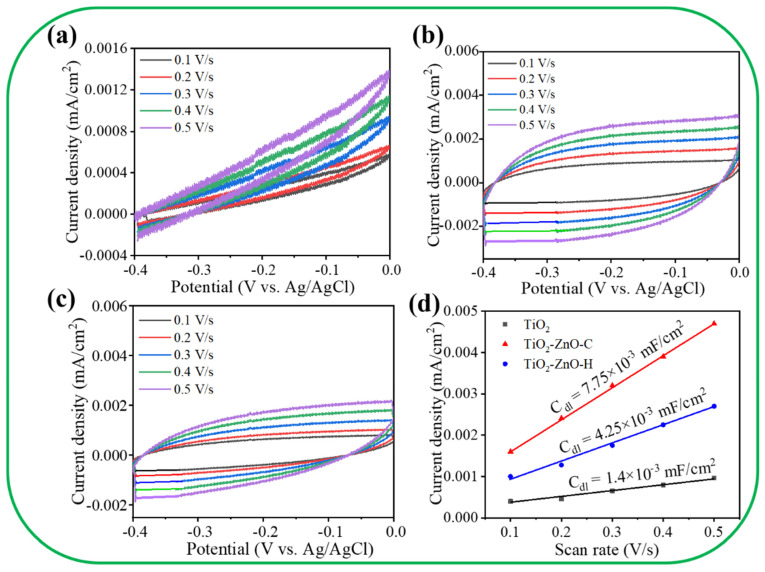
The current density vs. potential plots at various scan rates: (**a**) TiO_2_. (**b**) TiO_2_–ZnO–C. (**c**) TiO_2_–ZnO–H. (**d**) Current density vs. scan rate plot of various samples.

Figure 7a–c presents cyclic voltammogram (CV) curves at various scan rates for various samples. Figure 7d displays the electrochemical double-layer capacitance (C_dl_) values of various photoelectrodes calculated from the slope of the corresponding current density versus scan rate (*v*) curves according to the equation: capacitive current ∆j = *v* C_dl_ for a middle potential of −0.2 V [27]. For comparison, the result of the pristine TiO_2_ was included. Furthermore, C_dl_ is positively proportional to the electrochemical surface area (ECSA). The ECSA size of the as-fabricated photoanodes was estimated from their C_dl_ values [28]. The C_dl_ values of the TiO_2_ and TiO_2_–ZnO–H photoelectrodes were 1.4 × 10^−3^ and 4.25 × 10^−3^ mF/cm^2^, respectively. Remarkably, the C_dl_ value of the TiO_2_–ZnO–C electrode was 7.75 × 10^−3^ mF/cm^2^, which is approximately 1.8 and 5.5 times higher than the C_dl_ values of TiO_2_–ZnO–H and TiO_2_, respectively. A larger ECSA provided more active sites for the interface reaction between the as-fabricated photoelectrodes and the electrolyte. The excellent charge transfer behavior and high surface area were attributed to the large ECSA of the nanomaterials. The ZnO/V_2_O_5_ heterogeneous structure had a higher ECSA than pristine ZnO did; thus, the PEC performance of ZnO/V_2_O_5_ was superior to that of ZnO [29]. The high ECSA of TiO_2_–ZnO–C exposed a high number of surface active sites, which enabled the maintenance of a sufficient electrochemical reaction between the TiO_2_–ZnO–C nanostructures and electrolyte ions in this study. This result revealed that the morphology and crystal quality of the decorated ZnO crystals on the TiO_2_ nanorod template influenced the C_dl_ values and ECSA, which in turn affected the photoactive performance.

**Figure 8:**
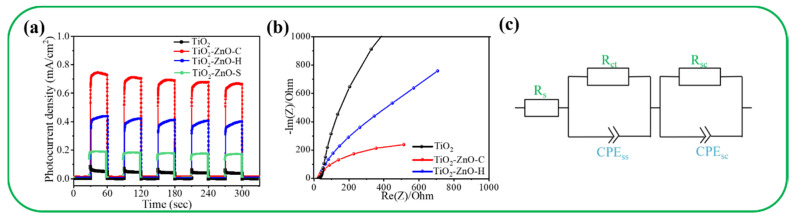
(**a**)Transient photocurrent density vs. time curves of various samples under on/off irradiation. (**b**) Nyquist plots of the TiO_2_ (black), TiO_2_–ZnO–C (red), and TiO_2_–ZnO–H (blue). (**c**) Possible equivalent circuit model used to evaluate the R_ct_ values of various composites.

Figure 8a displays the transient photocurrent responses of various samples under chopping light irradiation at 1.1 V. All the nanorod-based photoelectrodes demonstrated a visible photoresponse ability. Figure 8a indicates that the photocurrent density of the samples was stable, and the samples exhibited excellent reproducibility. The saturated photocurrent densities of the pristine TiO_2_ and TiO_2_–ZnO–S photoelectrodes were approximately 0.06 and 0.2 mA/cm^2^. The TiO_2_–ZnO–C photoelectrode exhibited the highest saturated photocurrent density of 0.75 mA/cm^2^, which was approximately 12 times that of TiO_2_. The TiO_2_–ZnO–H photoelectrode exhibited a saturated photocurrent density of approximately 0.42 mA/cm^2^, which was approximately seven times that of pristine TiO_2_. Notably, the ZnO seed layer coated TiO_2_ showed markedly lower photoresponse performance than the composites formed after the further decoration of ZnO crystals via chemical solution routes. The TiO_2_–ZnO-C composite photoelectrode exhibited superior photoresponse performance to the other photoelectrodes. The aforementioned result is similar to that obtained in a previous study for a WO_3_ nanosheet-decorated CdS nanorod composite, which revealed substantially improved PEC performance to pristine CdS because of the improved photogenerated charge separation ability in the heterogeneous system [30]. Figure 8b displays the Nyquist plots of the TiO_2_, TiO_2_–ZnO–C, and TiO_2_–ZnO–H photoelectrodes under irradiation. The TiO_2_–ZnO–C photoelectrode had the smallest arc radius among all the photoelectrodes, which indicated that the TiO_2_–ZnO–C photoelectrode had the lowest charge transfer resistance [31]. Figure 8c presents the possible equivalent circuit for the TiO_2_–ZnO composite photoelectrodes in the Nyquist plot. In Figure 8c, R_sc_ is the solution resistance, and R_ct_ is the surface state resistance that is related to the charge transfer from the valence band or conduction band to the semiconductor electrode surface [32]. The parameters CPE_ss_ and CPE_sc_ are constant-phase elements for the electrolyte–electrode interface and electrode surface, respectively [33]. The R_ct_ values of various composite photoelectrodes were evaluated by fitting the Nyquist plot under the proposed equivalent circuit mode. The TiO_2_-ZnO-C and TiO_2_-ZnO-H photoelectrodes exhibited R_ct_ values of 352 and 559 Ω, respectively. Notably, the R_ct_ of the TiO_2_ photoelectrode is high and reaches 3507 Ω. The results revealed that the ZnO nanodisks in TiO_2_–ZnO–C provided superior photoactive performance to those in TiO_2_–ZnO–H.

**Figure 9:**
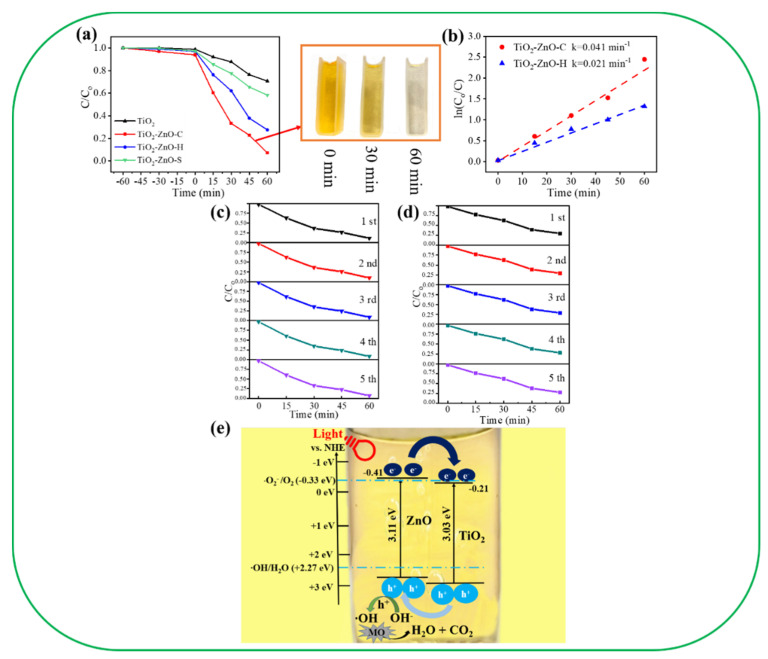
(**a**) Photodegradation level of the methyl orange (MO) solution in the presence of various samples. The inset show the discoloration of the MO solution containing the TiO_2_–ZnO–C under various irradiation durations. (**b**) Kinetic reaction rate k for the degradation of the MO solution with the TiO_2_–ZnO–C, and TiO_2_–ZnO–H. (**c**) Recycling photodegradation tests of the MO solution containing TiO_2_–ZnO–C. (**d**) Recycling photodegradation tests of the MO solution containing TiO_2_–ZnO–H. (**e**) A possible band alignment of the TiO_2_–ZnO and photodegradation reaction of the TiO_2_–ZnO towards the MO dye.

Figure 9a displays the relative concentration (C/Co) of the MO solution vs. irradiation time plots for various photocatalysts. Prior to irradiation, the adsorption/desorption equilibrium of the MO solution with various photocatalysts in the dark condition was established. In the presence of the pristine TiO_2_ and TiO_2_–ZnO–S photocatalysts, the degradation of the MO solution only reached 29.4% and 42.1% after 60 min of light irradiation. In contrast, the photodegradation of the MO solution is significantly increased to 92.4% with the TiO_2_–ZnO–C photocatalyst, whereas the TiO_2_-ZnO-H photocatalyst exhibited moderate photocatalytic activity and photodegraded the MO solution at 72.4%. The inset in Figure 9a also presents the discoloration of MO solution containing TiO_2_–ZnO–C at various irradiation durations. Figure 9b indicates that the TiO_2_–ZnO–C photocatalyst had the highest rate constant (k = 0.041 min^−1^), which was approximately 1.96 times that of TiO_2_–ZnO–H (k = 0.021 min^−1^) according to the pseudo-first-order kinetics at low initial concentrations. Furthermore, cycling experiments were performed to evaluate the stability and reusability of the TiO_2_–ZnO–C and TiO_2_–ZnO–H photocatalysts (Figure 9c,d, respectively). After five reuse tests, the TiO_2_–ZnO–C and TiO_2_–ZnO–H photoelectrodes exhibited high stability and reusability. Approximately 88.9% and 70.4% photodegradation of the MO solution was observed for the TiO_2_–ZnO–C and TiO_2_–ZnO–H photoelectrodes, respectively. This result indicates that the synthesized TiO_2_–ZnO–C and TiO_2_–ZnO–H composite photocatalysts have excellent stability and reusability and are promising for photocatalysis applications. The possible photodegradation mechanism of the TiO_2_–ZnO composite photocatalyst is illustrated in Figure 9e. In the TiO_2_–ZnO composite system, the conduction band of TiO_2_ was located at −0.21 eV and that of ZnO was located at approximately −0.41 eV [19]. Figure 9e indicates that the constructed TiO_2_/ZnO system exhibited a staggered type-II band alignment configuration, which is similar to that of the TiO_2_ nanowire/ZnO nanoparticle system [20]. Interband transitions in ZnO and TiO_2_ were speculated to have occurred during light irradiation. Because of the various positions of the valence and conduction bands of TiO_2_ and ZnO, the recombination of the photoinduced carriers in the composite system was suppressed according to the following reaction: TiO_2_–ZnO composite nanorods + *hv* (charge separation)→TiO_2_ nanorod template (e^−^) + ZnO coverage layer (h^+^). Moreover, the root of the TiO_2_ template without the coverage of ZnO will also produce photoexcited e^−^ and h^+^. Notably, the oxidation(⋅O_2_^−^/O_2_) and reduction (⋅OH/H_2_O) potentials were −0.33 and +2.27 eV (vs. NHE), respectively [34], which indicated that the electrons that accumulated on the TiO_2_ conduction band in the composite system or the pristine TiO_2_ section were not involved in the photodegradation process of the MO dye through the formation of ⋅O_2_^−^ radicals. By contrast, the highly oxidative holes on the ZnO valence band were involved in the reactions with bounded hydroxide species to produce ⋅OH radicals according to the reaction OH^−^ + h^+^→ ⋅OH. The ⋅OH radicals further reacted with MO molecules to form CO_2_ and H_2_O. Thus, the h^+^ and ⋅OH radicals played a crucial role in photodegrading the adsorbed MO molecules, which led to the formation of a strong photocatalyst for the TiO_2_–ZnO heterojunction. Comparatively, from the PEC and photodegradation results, the free h^+^ involved in photocatalytic reaction contributed from the pristine TiO_2_ section of the composite system is relatively low. The heterogeneous effect on the charge separation efficiency is the predominant role of the improved photodegradation performance of the TiO_2_–ZnO composite. Notably, TiO_2_–ZnO–C demonstrated a higher photodegradation efficiency for MO dyes than TiO_2_–ZnO–H did. This result was attributed to the several advantages of the decorated ZnO nanodisks. The ZnO nanodisks exhibited higher crystalline quality than the ZnO filaments did. Moreover, the ZnO nanodisks exhibited a larger surface area than the ZnO filaments did, which resulted in increases in the dye adsorption, sunlight utilization capability, and photocatalytic activity of the exposed facets in TiO_2_–ZnO–C.

## 4. Conclusions

In this study, 1D TiO_2_ and 2D ZnO nanocomposites were fabricated using the sputtering-grown ZnO-seed-layer-assisted method and various chemical ZnO synthesis methods. The chemical synthesis method affects the morphology and crystal quality of the 2D ZnO nanostructures on TiO_2_ nanorod templates. The results for PEC, EIS, ECSA, and the hydrophilic property revealed that the ZnO nanodisks caused the TiO_2_–ZnO composite to have higher photoactivity than the ZnO filaments did. The TiO_2_–ZnO system exhibited a type-II band alignment configuration, which explains why the TiO_2_–ZnO composite had a higher photoactivity than pristine TiO_2_. The experimental results indicated that the TiO_2_–ZnO–C composite structure had superior photophysiochemical properties to the TiO_2_–ZnO–H structure. This result was associated with the superior surface area and crystalline quality of the 2D ZnO crystals in TiO_2_–ZnO–C.
